# Multiple cross displacement amplification-a more applicable technique in detecting *Pseudomonas aeruginosa* of ventilator-associated pneumonia (VAP)

**DOI:** 10.1186/s13054-020-03003-4

**Published:** 2020-06-08

**Authors:** Juxiang Wang, Huimin Chen, Xiaomin Lin, Chengyi Ji, Bin Chen

**Affiliations:** 1 Department of Intensive Care Unit, Xiamen Cardiovascular Hospital, Xiamen University, Xiamen, Fujian China; 2 Department of Intensive Care Unit, The Third Hospital of Xiamen, Xiamen, Fujian China; 3 Department of Intensive Care Unit, Xiamen Branch, Zhongshan Hospital, Fudan University, Xiamen, Fujian China; 4 Department of Healthcare, Xiamen Port Clinic of Xiamen Customs, Xiamen, Fujian China

**Keywords:** *Pseudomonas aeruginosa*, Multiple cross displacement amplification, Ventilated-associated pneumonia, Bronchoalveolar lavage fluid, Limit of detection

## Abstract

**Background:**

Early and rapid identification of *Pseudomonas aeruginosa* (*P. aeruginosa*) in patients with suspected ventilator-associated pneumonia (VAP) provides theoretical clinical advantages in therapeutic optimization strategies.

**Methods:**

The *P. aeruginosa-*multiple cross displacement amplification (*PA-MCDA)* assay was conducted at an isothermal temperature during the amplification stage, and products were visually detected by color changes. The entire process was completed within 1 h. A total of 77 strains, including *P. aeruginosa* species and various other species of non-*P. aeruginosa*, were used to evaluate *PA*-MCDA assays. Bronchoalveolar lavage fluid (BALF) of suspected VAP patients was examined by the MCDA assay.

**Results:**

The MCDA assay exhibited a 100% analytical specificity in detecting *PA* from all 77 strains, and the limit of detection was as low as 100 fg DNA per reaction. A temperature of 65 °C was recommended as standard during the amplification stage. The agreement between PA-MCDA and bacteria culture was 91.18% (*κ* = 0.787; *p = 0.000*) in the identification of *P. aeruginosa* in BALF from suspected VAP. The PA-MCDA assay showed values of 92.31%, 90.78%, 77.41%, and 97.18% for sensitivity, specificity, positive predictive value, and negative predictive value, respectively. PA-MCDA had a higher detective rate of *P. aeruginosa* than bacteria culture in patients with antipseudomonal therapy.

**Conclusions:**

The instrument-free platform of the MCDA assay makes it a simple, rapid, and applicable procedure for “on-site” diagnosis and point-of-care testing for the presence of *P. aeruginosa* without the need for specific bacterial culture.

## Background

Ventilator-associated pneumonia (VAP) develops in intensive care unit (ICU) patients that have been mechanically ventilated for at least 48 h [1]. The infection rate was related to disease severity and the degree of organ failure [2]. Further, the EU-VAP study [3] identified that the overall incidence of VAP was 18.3 episodes per 1000 ventilator-days. Moreover, *Pseudomonas aeruginosa* (*P. aeruginosa*) and *Staphylococcus aureus* were the most frequently isolated pathogens in patients with VAP. In a multicenter study [4], *P. aeruginosa*, *Acinetobacter baumannii*, and *Klebsiella pneumonia* were found as the most frequent bacteria (97/212, 45.8%) in VAP patients. In addition, VAP induced a more prolonged need for mechanical ventilation, number of ICU stays and hospitalization, far worse outcomes, and increased associated healthcare costs [5, 6].

Rapid completion of antibiotic administration might decrease the incidence of subsequent organ dysfunction and could be associated with a lower risk-adjusted in-hospital mortality rate [7]*.* The conventional detection of *P. aeruginosa* in the clinical setting was generally achieved by growing the target pathogen on agar plate surfaces and cultures [8]. Although the culture-based technique is reliable, the time required for conducting it is at least for a period of 48 h [9]. Furthermore, published guidelines recommended an initial empiric combinatorial coverage with antibiotics targeted to Gram-negative and Gram-positive bacteria methicillin-resistant *Staphylococcus aureus* (MRSA) in the setting of high-risk VAP patients prior to obtaining culture results [1]. Inappropriate initial anti-microbial therapy and antibiotic exposure attributed to antibiotic resistance [10, 11] were associated with increased in-hospital mortality rates [12]. Rapid and accurate identification of the suspected pathogens was thus warranted to provide the potential to maximize the administration of appropriate specific antibiotic and possibly avoid a need for empiric broad-spectrum antibiotic therapy. Rapidly excluding some specific and common pathogens would be possible to avoid unnecessary antibiotic exposure and minimize some undesirable consequences [13].

More recently, multiple cross displacement amplification (MCDA, Chinese IP Office Patent Application CN201510280765.X), auto-cycling, and strand displacement DNA synthesis were devised and validated as a possible replacement for PCR-based assays and applicability in the detection of specific nucleic-acid sequences [14, 15]. The assay employed an isothermal temperature during amplification, and the products were visually detected by color changes. The entire process is completed in 1 h and benefits from being an instrument-free, simple, and practical procedure for “on-site” diagnosis and point-of-care testing. The current study is the first to report the application of the novel MCDA assay to rapidly detect the target pathogen, *P. aeruginosa*.

## Methods

### PA-MCDA assay primer design

**Fig. 1:**
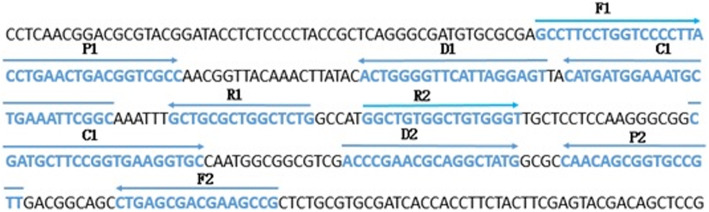
Schematic depiction of the primer sequences and positions for MCDA. The location and nucleotide sequence of the *P. aeruginosa* oprL gene that assisted in designing MCDA primers. The primer site sequences are underlined. Right and left arrows indicate sense and complementary sequences that were used in the assay

Based on the mechanism of MCDA, a set of MCDA primers used for *P. aeruginosa* detection was designed that targeted the *oprL* gene, which encodes l-lipoprotein. The details of MCDA primers used in the report are shown in Fig. 1 and Table 1. The primers were commercially synthesized and purified by Tsingke (Beijing, China).

**Table 1:** Primers used for multiple cross displacement amplification in this study

Primers	Sequences (5′-3′)	Length
CP1	GCCGAATTTCAGCATTTCCATCATG-CCTGAACTGACGGTCGCC	43mer
CP2	CGATGCTTCCGGTGAAGGTGC-AACGGCACCGCTGTTG	37mer
F1	GCCTTCCTGGTCCCCTTA	18 nt
F2	CGGCTTCGTCGCTCAG	16 nt
C1	GCCGAATTTCAGCATTTCCATCATG	25 nt
C2	CGATGCTTCCGGTGAAGGTGC	21 nt
D1	ACTCCTAATGAACCCCAGT	19 nt
D2	ACCCGAACGCAGGCTATG	18 nt
R1	CAGAGCCAGCGCAGCA	16 nt
R2	GGCTGTGGCTGTGGGT	16 nt
P1	CCTGAACTGACGGTCGCC	18 nt
P2	AACGGCACCGCTGTTG	16 nt

### *PA-*MCDA reactions

MCDA reactions were performed in a one-step reaction in a 25-μl mixture containing 12.5 μl 2× the supplied buffer (Beijing Hai Tai Zheng Yuan Technology Co., Ltd.); 0.1 μl each of the displacement primers F1 and F2; 0.2 μl each of the amplification primers C1, C2, R1, R2, D1, and D2; 0.4 μl each of the cross primers CP1 and CP2; 1 μl (8 U) of *Bst* 2.0 DNA polymerase; 1 μl of the DNA template; and 0.8 μl of the colorimetric indicator. Moreover, negative control mixtures contained 10 ng of the *Staphylococcus aureus* and *Klebsiella pneumoniae* genomic templates, and blank control mixtures contained 1 μl of double-distilled water (DW). To evaluate the feasibility of the MCDA primer set that was designed to detect *P. aeruginosa*, we initially conducted the MCDA reactions at 63 °C for 45 min and terminated the MCDA reaction by heating at 85 °C for 5 min. Then, the optimal amplification temperature of the MCDA primer set was examined at fixed temperatures from 59 to 68 °C at steps of 1 °C intervals. In particular, MCDA products were detected using a colorimetric indicator and agarose gel electrophoresis.

### Bacterial strains and genomic template preparation

A total of 124 bacterial strains and 14 fungi of positive culture were isolated in the clinical microorganism laboratory of the Third Hospital of Xiamen from 26 June to 26 July, 2017. The bacterial strains’ list of standard culture is detailed in Additional file 1*.* The positive bacterial strains including 6/124 (4.84%) polymicrobial growth and 32/124 (22.81%) multidrug-resistant (MDR) strains were isolated from 118 clinical samples in which the tracheal aspirate and BALF were in 38 cases, the secretion and drainage in 33 cases, the blood in 16 cases, and urine, feces, catheter, and other samples in 31 cases. We chose top 13 bacteria strains and 77 samples to design the PA-MCDA reactions (Table 2). The bacteria strains were identified by conventional cultivation method, automatic bacterial identification system (VITEK 2, Bio-Merieux, France) and stored in a 15% (w/v) glycerol broth at − 70 °C. After refreshing the culture three times on a nutrient agar plate at 37 °C, the genomic templates were then extracted from all cultured strains using DNA extraction kits (Qiagen Co., Ltd., Beijing, China) and subsequently tested with an ultraviolet spectrophotometer and stored under − 20 °C before use.

**Table 2:** List of bacterial strains

Bacteria	Strains	Bacteria	Strains
*Pseudomonas aeruginosa*	17	*Streptococcus agalactiae*	5
*Escherichia coli*	5	*Enterococcus faecalis*	5
*Staphylococcus aureus*	5	*Salmonella typhimurium*	5
*Acinetobacter baumannii*	5	*Klebsiella pneumoniae*	5
*Staphylococcus epidermidis*	5	*Enterobacter cloacae*	5
*Staphylococcus capitis*	5	*Stenotrophomonas maltophilia*	5
*Streptococcus pyogenes*	5		

A total of 77 bacterial strains, which included 17 PA and 60 non-PA strains, were identified by standard culture by the clinical microorganism laboratory of the Third Hospital of Xiamen

### Specificity of the *PA*-MCDA assay

To evaluate the analytical specificity of the *PA*-MCDA assay, MCDA reactions were conducted under conditions that were described above with the 77 *P. aeruginosa* and non-*P. aeruginosa* pure genomic templates that were derived from all pure bacterial strains.

### Sensitivity of the *PA*-MCDA assay

The genomic templates of *P. aeruginosa* were serially diluted (10 ng, 1 ng, 100 pg, 10 pg, 1 pg, 100 fg, 10 fg, and 1 fg per microliter) with the intent of verifying the limit of detection (LoD), and 1 μl of each serial dilution was then added to the MCDA reaction mixtures. The LoD of the MCDA assay was confirmed by the genomic DNA concentration of the template. MCDA results were detected using a colorimetric indicator, malachite green, MG (Beijing Hai Tai Zheng Yuan Technology Co., Ltd.) and 2.5% agarose gel electrophoresis.

### Verification of the *PA*-MCDA assay

This study was conducted in the 30-bed ICU of The Third Hospital of Xiamen in Fujian province, which is a 1200-bed hospital in China. A total of 102 patients enrolled in this study, who were with suspected VAP from 1 January 2018 to 14 March 2020. Patients satisfied two or more of the following criteria: fever > 38.5 °C, leukocytosis > 10^9^/L or leukopenia < 4 × 10^8^/L, purulent tracheobronchial secretions, and a new or persistent infiltrate on chest radiography. The clinical  data were recorded, i.e, demographic characteristics, indication(s) for ICU admission, prior antimicrobial therapy within 2 months before VAP, duration of mechanical ventilation before VAP, clinical pulmonary infection score (CPIS [16]) including temperature, blood leukocytes, tracheal secretions, oxygenation, pulmonary radiography, biochemical and hematological  routine tests.

The BALF were abstracted in one bottle, following which, one half (5 ml) processed for standard culture by the clinical microorganism laboratory of the Third Hospital of Xiamen and the other 5 ml stored at − 70 °C until the time of DNA extraction. BALF was plated on chocolate, sheep blood, and MacConkey agar plates and incubated for 48–72 h according to routine clinical protocol. The DNA extraction from BALF method was described before. In this procedure, 1 μl of the extracted DNA template of the BALF specimen was added to the *PA-*MCDA assay and the reactions were performed at an optimal amplification temperature for 45 min. The products were detected by a color change and compared to the results of a standard clinical culture, which was blinded to the research investigators. This study was approved by the local ethics committee of the Third Hospital of Xiamen and performed according to the ethical standards of the latest revision of the Declaration of Helsinki. Written and informed consent was obtained from family members or the appropriate responsible parties.

### Statistical analysis

Continuous variables of patients’ characteristics were reported as the means ± standard deviations (SD) or the medians (interquartile ranges (IQR)), and categorical variables were reported as numbers (%). The accuracy of the PA-MCDA assay was compared with the microbiological culture in a cross-sectional analysis. *P value* < 0.05 was considered significant. Statistical analysis was performed using SPSS version 20.0 for Windows (SPSS Inc., Chicago, IL, USA).

## Results

### Successful establishment of the PA-MCDA assay

**Fig. 2:**
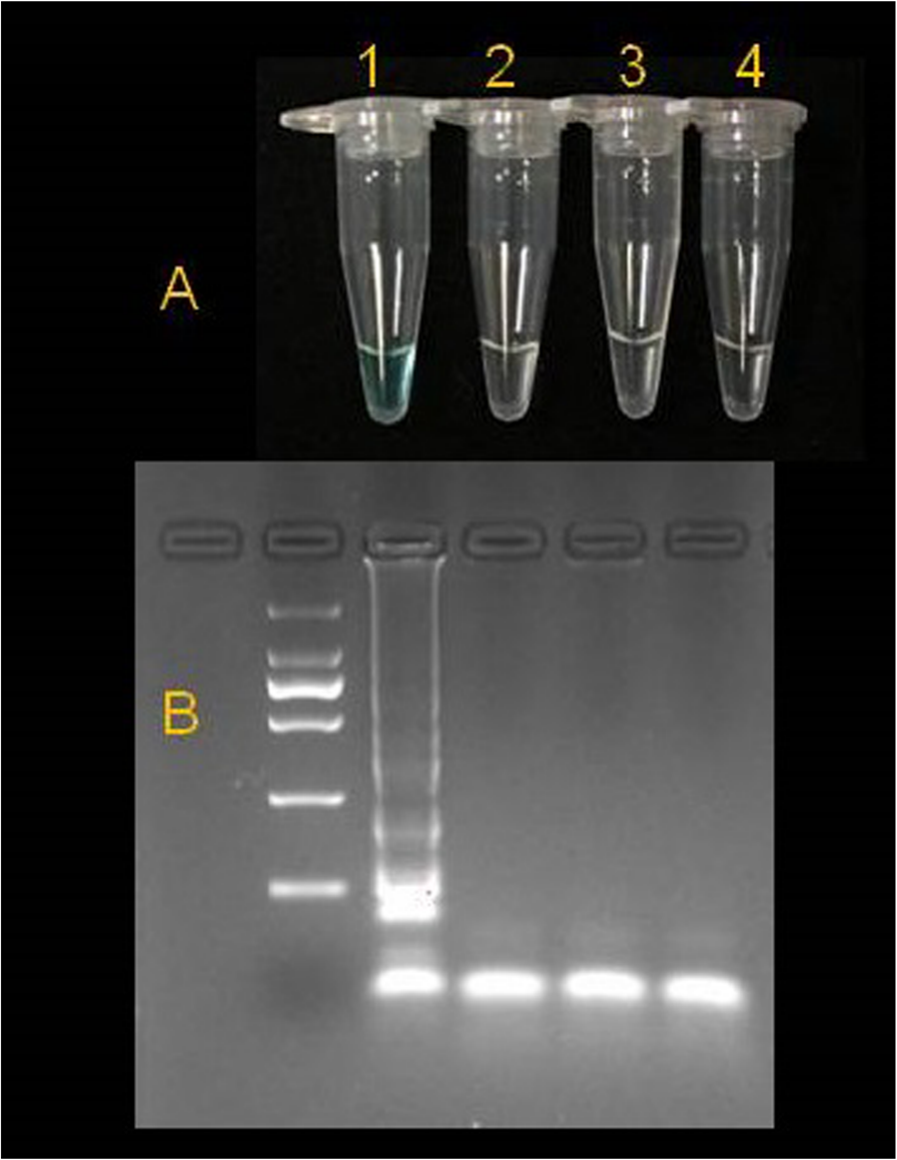
Confirmation and detection of products. **a** The color change was seen in MCDA tubes: tube 1 was the positive amplification of *P. aeruginosa*, the green color was observed directly; tubes 2 and 3 were the negative amplifications of *S. aureus* and *K. pneumoniae* respectively; tube 4 was the negative amplification of the control (no DNA); tubes 2, 3, and 4 remained colorless. **b** 2.5% agarose gel electrophoresis was applied to MCDA; lane 0: DL 100-bp DNA marker; lane 1: positive MCDA products of *P. aeruginosa*; lanes 2 and 3: negative products of *S. aureus* and *K. pneumoniae* respectively; and lane 4, negative control (no DNA)

To verify the feasibility of *PA*-MCDA primers, the MCDA reactions were initially carried out in the presence or absence of genomic DNA templates within 45 min at a constant temperature of 63 °C [14]. A color shift of positive amplification in *PA*-MCDA tubes was directly observed to change from one of colorless to one of green, while the negative control tube remained colorless by the naked eye (Fig. 2a). The positive MCDA products were seen as ladder-like patterned bands on ethidium bromide-stained 2.5% agarose gels that were resolved by electrophoresis; however, these were not seen in the *Staphylococcus aureus*, *Klebsiella pneumonia*, or blank control (Fig. 2b). Hence, the designed MCDA primer was a good candidate to establish the MCDA methodology for detecting *P. aeruginosa*.

### Optimizing the temperature for the PA-MCDA assay

**Fig. 3:**
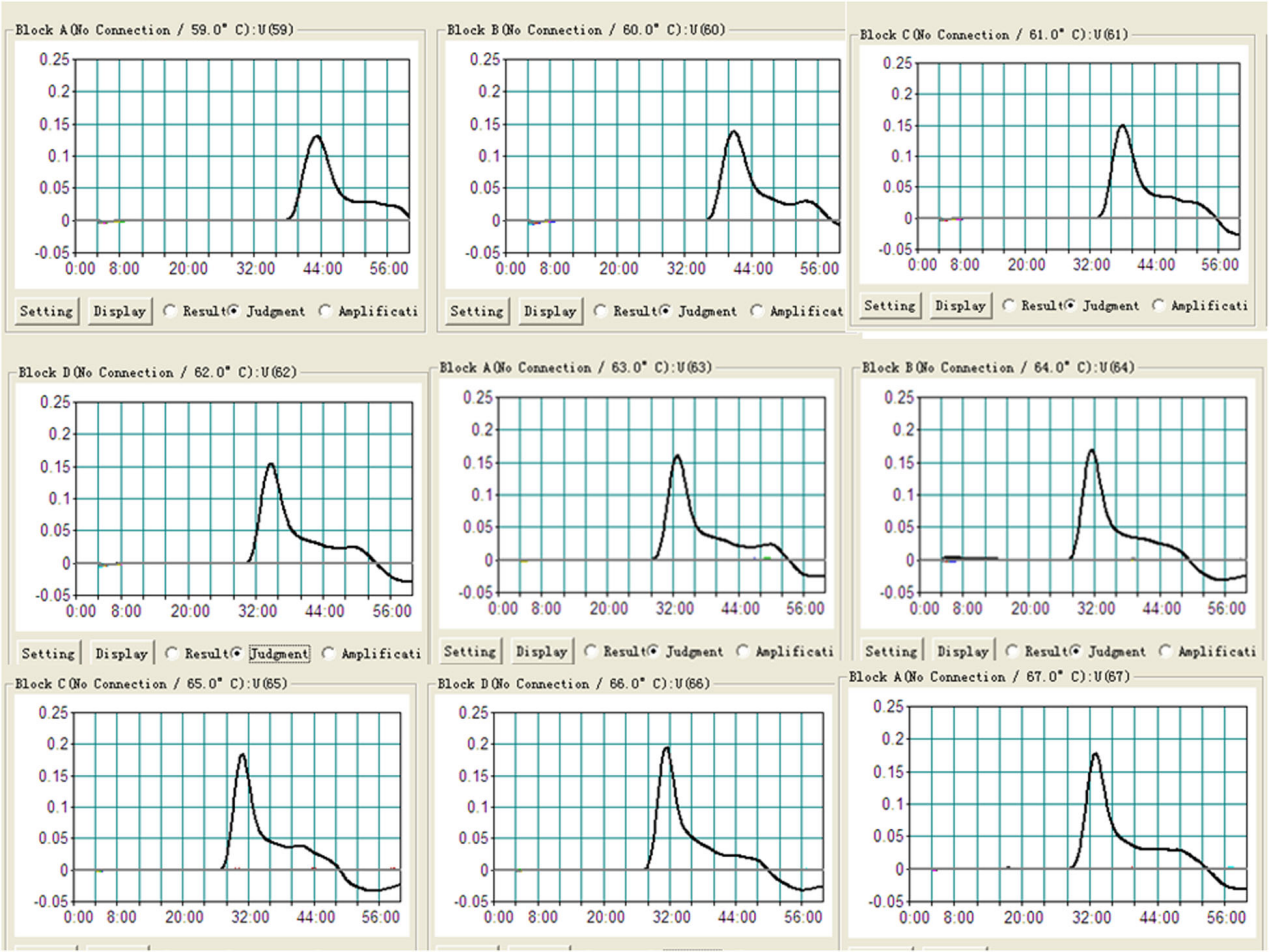
Optimal reaction temperature for the PA-MCDA primer assay. MCDA reactions when detecting the *P. aeruginosa* gene were monitored by real-time measurement of turbidity. A turbidity of > 0.1 was considered positive. Nine kinetic graphs were obtained at various temperatures (59–67 °C, at 1 °C intervals) with *P. aeruginosa* DNA at a concentration of 1 ng per tube. The graphs showed that 65 °C was an optimal temperature for amplification

To confirm the optimal reaction temperature for the *PA*-MCDA assay, the *P. aeruginosa* strain was used as a positive control at a concentration of 1 ng per tube and the MCDA amplifications were monitored by a real-time turbidity technique. Performing the *PA*-MCDA assay at temperatures that ranged from 59 to 67 °C at 1 °C increments verified that 65 °C was an optimal temperature for amplification—with a faster amplification procedure obtained from assay temperatures of 65 °C (Fig. 3).

### Specificity and sensitivity for *P. aeruginosa* detection by MCDA assays

**Fig. 4:**
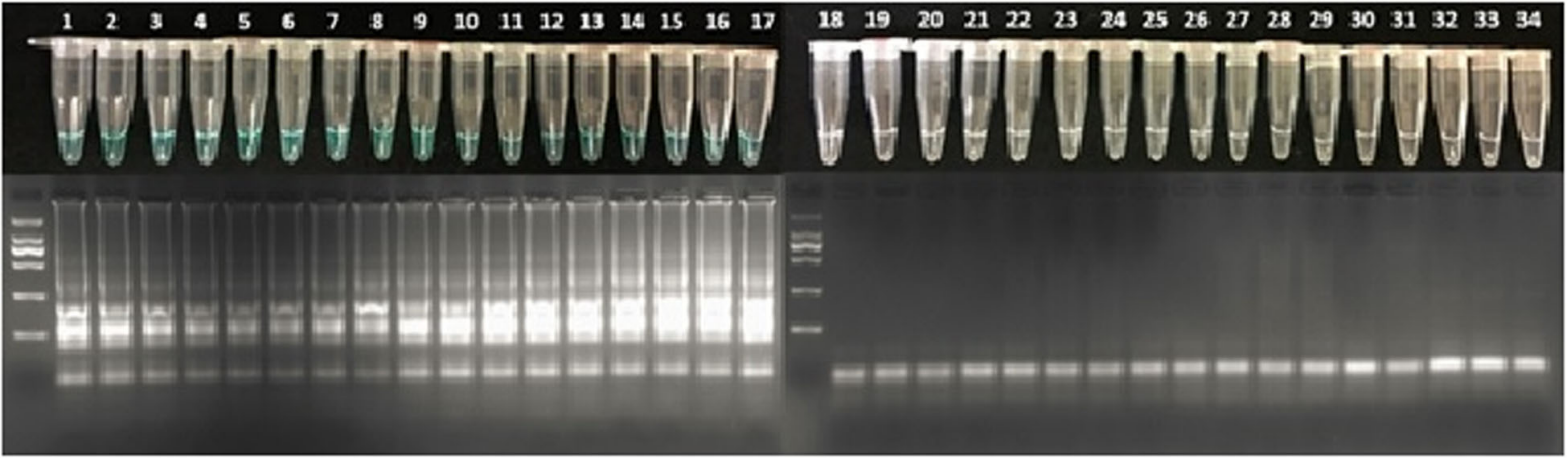
Specificity for *P. aeruginosa* detection by MCDA assays. Of all 77 pure genomic templates, only the genomic DNAs from the *P. aeruginosa* strains generated positive results. The color shift in the PA-MCDA tubes (tubes 1–17) was directly observed as a green color. A gray color was seen in tubes 18–34, in which 18–21 were *E. coli*. Tubes 22–23 were *S. aureus*, of which tubes 24–25 were *A. baumannii* and tubes 26–34 were respectively *S. epidermidis*, *S. capitis*, *Streptococcus pyogenes*, *S. agalactiae*, *E. faecalis*, *S. typhimurium*, *K. pneumoniae*, *E. cloacae*, and *S. maltophilia.* A 2% agarose gel electrophoresis assay was applied to detect *P. aeruginosa* MCDA; lane 0, DL 100-bp DNA marker; lanes 1–17 were positive MCDA products that corresponded to *P. aeruginosa* tubes 1–17; and lanes 18–34 were negative and corresponded to tube 18–34 respectively

When genomic templates were used in MCDA assays, only the genomic DNAs that were isolated from the *P. aeruginosa* strains (tubes 1 to 17) generated positive results. Genomic templates from all non-*P. aeruginosa* strains (tubes 18 to 34) did not provide production of detectable amplification products (Fig. 4). The color change was observed in positive MCDA tubes (tubes 1 to 17), and a ladder-like pattern was seen on an ethidium bromide-stained 2.5% agarose gel via electrophoresis resolution. These were not seen in negative tubes (Fig. 4).

**Fig. 5:**
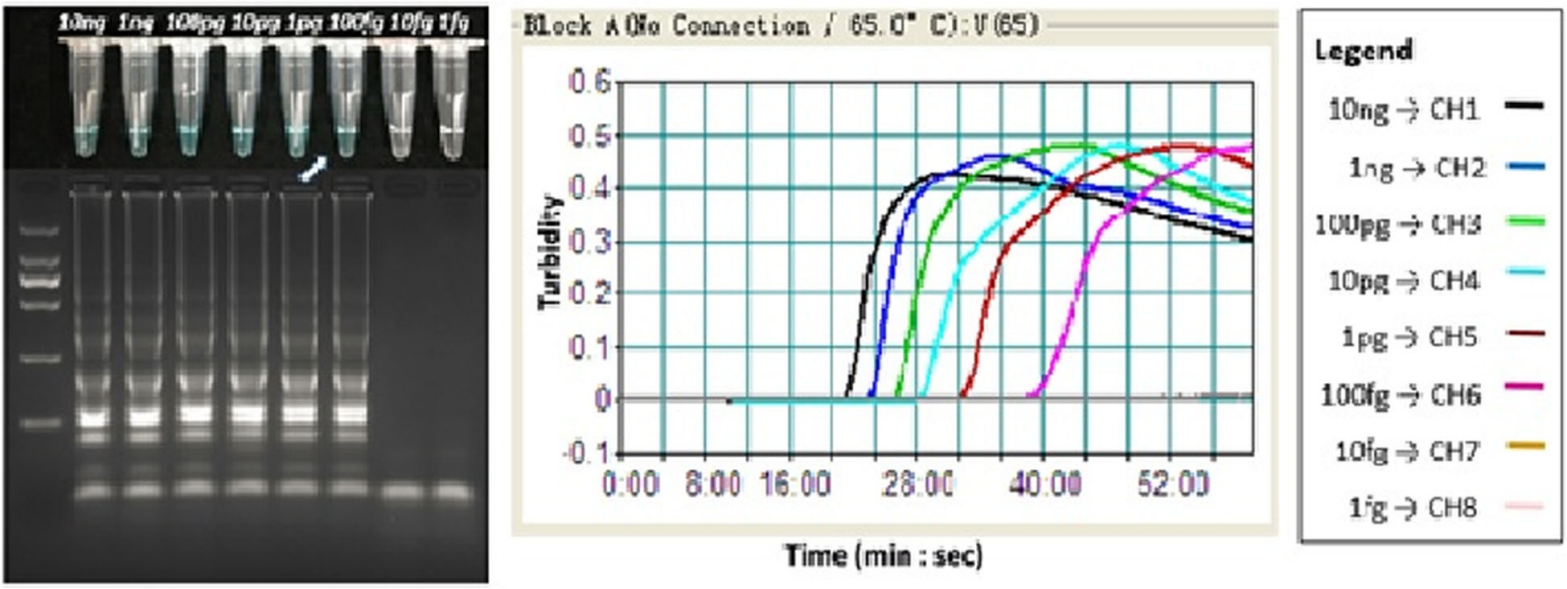
Sensitivity of the MCDA assays using serially diluted *P. aeruginosa* genomic DNA**. a***P. aeruginosa* genomic DNA was serially diluted to 10 ng, 1 ng, 100 pg, 10 pg, 1 pg, 100 fg, 10 fg, and 1 fg per microliter. When the dilution was more than 100 fg/uL, the green color by MG and the laddering *P. aeruginosa* pattern by agarose gel electrophoresis were directly observed. The LoD of the PA MCDA assay was as low as 100 fg per microliter (white arrow). **b** Real-time turbidity was applied to analyze the amplification products. Genomic DNA levels > 100 fg per reaction produced positive reactions

Serial dilution of the *P. aeruginosa* genomic DNA (10 ng, 1 ng, 100 pg, 10 pg, 1 pg, 100 fg, 10 fg, and 1 fg per microliter) was used in MCDA assays. When the dilution exceeded 100 fg/uL, the green color by MG, the ladder pattern by agarose gel electrophoresis, and the positive reactions on real-time turbidity products were all directly observed (Fig. 5a and b). It indicated that the LoD and the sensitivity of the PA-MCDA assay were 100 fg genomic templates per reaction.

### Application of PA-MCDA to clinical samples

The MCDA assay was used to examine 102 BALF from patients who were suspected of presenting with VAP. Demographic and clinical characteristics of our patients are shown in Table 3. The median duration of mechanical ventilation was 7 days (IQR 4.00–32.50) before suspected VAP onset, and 97 patients had received antibiotics within 2 months before suspected VAP. The clinical diagnosis of VAP was given by physician judgment with respect to particular patients or special clinical situations combination with culture results. A total of 94 positive bacteria results were detected by conventional culture in 82/102 (80.39%) BALFs in which polymicrobial growth was 12/102 (11.76%) and polymicrobial included *P. aeruginosa* was 4/102 (3.92%). Bacterial strains list of BALF by standard culture is detailed in Additional file 2. After extracting DNA from these BALF specimens and adding 1 μl of the DNA template to the *PA-*MCDA assay, the reactions were carried out at 65 °C for 45 min and the results were compared to those of the standard culture. The *P. aeruginosa* was detected in 26 samples by microbiological culture and in 31 samples by PA-MCDA. Two methods unanimously detected 24 *P. aeruginosa* strains while the MCDA detected 7 *P. aeruginosa* strains in culture-negative patients who received antipseudomonal therapy. The positive results of PA-MCDA and microbiological culture were 24/76 (31.58%) and 17/76 (22.37%) respectively in 76 patients who had received antipseudomonal therapy. In the cross-sectional analysis, the agreement between the tests was 91.18% (*κ* = 0.787; *p = 0.000*), likelihood ratio positive was 10.02, and the likelihood ratio negative was 0.08. The PA-MCDA assay showed values of 92.31%, 90.78%, 77.41%, and 97.18% for sensitivity, specificity, positive predictive value, and negative predictive value, respectively.

**Table 3:** Demographic, clinical, and biological characteristics of patients

	Total (*n* = 102)
**Characteristics at ICU admission**	
Male sex, *n* (%)	75 (73.5%)
Age (years), median (IQR)	54.62 ± 19.59, 57 (39.75–70.75)
Medical patients, *n* (%)	39 (38.2%)
Surgery patients, *n* (%)	63 (61.8%)
Prior antibiotics in 90 days, *n* (%)	61 (59.8%)
**Characteristics upon VAP onset**	
ICU stay (days), median (IQR)	37.87 ± 80.45, 8 (4.00–37.75)
Duration of MV* (days), median (IQR)	35.42 ± 80.05, 7 (4.00–32.50)
Septic shock, *n* (%)	57 (55.9%)
new or persistent infiltrate on chest radiography	72 (70.59%)
CPIS	6.59 ± 1.70, 7 (5.00–8.00)
CRP	84.59 ± 57.98, 69.45 (46.00–104.80)
PCT	17.34 ± 24.29, 10 (2.34–21.30)
**Antibiotics within 3 days**	
None	5 (4.9%)
Monotherapy	41 (40.2%)
Combination antibiotic therapy	56 (54.9)
covering PA	76 (74.5%)
Change of antibiotics, *n* (%)	45 (44.1%)
**Clinical diagnosis of VAP**	68 (66.7%)

*Duration of mechanical ventilation before suspected VAP

*CPIS* clinical pulmonary infection score

## Discussion

PA-MCDA reaction was performed with a set of 10 oligonucleotide primers, which specifically recognized 10 distinct sites on the target sequence, wherein the optimal temperature for amplification was 65 °C. In particular, a colorimetric indicator (malachite green, MG) had been applied and the color changed from colorless to a light green color when the reaction was positive. The specificity of the PA-MCDA assay was 100%, and the sensitivity achieved a level as low as 100 fg of the template. The entire procedure, including that of specimen processing (15 min), the isothermal reaction, and result reporting (45 min), could be completed in approximately 1 h. The agreement between PA-MCDA and bacteria culture was 91.18% (*κ* = 0.787; *p = 0.000*) in the identification of *P. aeruginosa* in BALF from suspected VAP. PA-MCDA had a higher detective rate of *P. aeruginosa* than bacteria culture in patients who had received antipseudomonal therapy.

A rapid, simple, and accurate detection method of pathogenic microorganisms was necessary for the timely administration of appropriate therapy and arriving at a time to discontinue unnecessary antibiotic(s). Delayed receipt of an appropriate antibiotic(s) was independently associated with poorer clinical and economic outcomes in patients with serious Gram-negative bacterial infections, regardless of any resistance status [9]. Molecular diagnostic assays, such as PCR-based methods (e.g., conventional PCR, real-time PCR [17], and PCR-Electro Spray Ionization MS (PCR/ESI-MS) [18]), permit more rapid detection of targeted bacterium by nucleic acid amplification and have been established and applied in the clinic. However, these PCR-based techniques have some shortcomings, which include the following: (i) the instrument used is extremely expensive, (ii) the diagnostic specificity is highly affected by the amplification conditions and the primer design, and (iii) use of these techniques indicates that PCR results require gel electrophoretic analysis or real-time analytical apparatus. Matrix-assisted laser desorption/ionization time of flight mass spectrometry (MALDI-TOF) [19] consistently decreased the time for successful identification of the pathogenic organism, shorten the period of time for administering an effective and optimal antibiotic, and thus arrive at improved patient outcomes. However, it also needs a bacterial culture and expensive instruments.

For diagnosis of VAP, not the standard culture but the MCDA assays are intended to supplant physician judgment respect to particular patients or special clinical situations. The PA-MCDA assay just represents a rapid, sensitive, and nearly instrument-free method of *P. aeruginosa* detection. Only a water bath or heat block was needed during the reaction stage, and the cost is about $8 per sample compared to standard culture which is $10 per sample. Due to MCDA being able to provide results within only 1 h, the clinician can save time to provide targeted therapy for patients, especially in the context of severe sepsis or septic shock patients. The positive test of PA-MCDA means there were *P. aeruginosa* in BALF; an initial narrow antibiotic therapy with antipseudomonal activity should be given in stable patients with suspected VAP in order to provide targeted therapy and reduce antibiotic exposure. The broad-spectrum empiric therapy with antipseudomonal activity needed to be given to patients with severe acute respiratory distress syndrome and profound (unstable) septic shock when the PA-MCDA were positive because the high sensitivity for PA-MCDA assays and the polymicrobial growth always existed. The negative test indicated that there were no *P. aeruginosa* growth or the number of *P. aeruginosa* was very below the 10^4^ cfu/ml in the BALFs as the high sensitivity of PA-MCDA assay [20]. The value was that empiric combination coverage for *P. aeruginosa* might not be necessary and the discontinuation of antibiotic therapy with antipseudomonal activity needed to be considered by clinicians [13]. Although the bacterium specific-MCDA assay cannot differentiate the infection from colonization for it cannot quantified the bacteria strains of BALF that is the methodology to diagnose VAP, the different time of color change of positive MCDA assay may relate to the amount of DNA templates of bacteria because its reaction products could be detected by real-time fluorescence and less time of positive reactions were produced in more specific-DNA templates [20]. Whether a threshold time of color change for MCDA reflects the quantitative or semiquantitative bacterium for clinical application needs a precise design and analysis. The PA-MCDA assay also detected 7 positive *P. aeruginosa*, while standard culture was negative in 76 patients who had received antipseudomonal therapy. The interpretation might suggest the MCDA approach had a higher sensitivity than standard culture [15, 21], or that the culture negativity might reflect the presence of active culture inhibitors in the samples [22], or that the *P. aeruginosa* had become non-viable before or even between the standard culture periods since the growth conditions had changed. Clinical factors should also be taken into account because they might alter the decision of whether to withhold or continue antibiotics. The ultimate clinical determination of VAP and pathogens, and regarding antibiotics application, was made by the physician in the light of each patient’s individual circumstances [13].

In order to use MCDA method to clinical application, this study also has limitations. The first is the bacterium-specific MCDA assay cannot differentiate the infection from colonization because it cannot provide the results of the quantitative bacterium of BALF. A more precise study will be designed recently to explore the relationship between a cutoff time for MCDA color change and the quantitative or semiquantitative bacterium in clinical samples. In addition, whether or not the assay could detect the target pathogen in other specimens such as blood, urine, or serous effusion needs further study. Consequently, both the negative and positive results of PA-MCDA cannot rule out the presence of other pathogens, Gram-positive or Gram-negative bacteria, or fungi, for polymicrobial growth always existed. MCDA cannot provide precise information to prescribe or withdrew pathogen-specific therapy [23] until more pathogen-specific MCDA and resistance-associated genes are designed [20]. We propose to assign some microorganism-specific MCDA assays and resistance-associated genes (e.g., the nfxB gene and the blaPER-1 gene) [18, 19, 24, 25] that are aligned to common pathogens in ICU, which would include *Staphylococcus aureus* [26], *Acinetobacter baumannii*, *Escherichia coli*, fungal species, and others in one template.

## Conclusions

The PA-MCDA assay for rapid detection of *P. aeruginosa*, which was based on the oprL gene, was successfully developed. This approach enabled its reaction products to be identified by the naked eye, and the assay established a high degree of both specificity and sensitivity for target template analysis. The PA-MCDA assay does not only have the benefit of a rapid, reliable, and nearly instrument-free procedure, but it can also differentiate *P. aeruginosa* from pure strains of bacteria and clinical specimens without the need for time-consuming bacterial culture, and its clinical significance needs further establishment.

## Supplementary information

**Additional file 1.** Bacterial strains list of standard culture.

**Additional file 2.** Bacterial strains list of BALF by standard culture.

## Data Availability

The dataset analyzed during the current study is available from the corresponding author on reasonable request.
