# Supplementary material for: Multiple cross displacement amplification-a more applicable technique in detecting Pseudomonas aeruginosa of ventilator-associated pneumonia (VAP)
Source: Crit Care. 2020 Jun 8;24:306. doi: 10.1186/s13054-020-03003-4 (PMC7276953; doi:10.1186/s13054-020-03003-4)
Supplement: Supplementary file 1 — Additional file 1. Bacterial strains list of standard culture. [file 13054_2020_3003_MOESM1_ESM.docx]

### Additional file 1 –Bacterial strains list of standard culture

| Bacteria | Strains(MDR) | Frequency (%) |
| --- | --- | --- |
| *Pseudomonas. aeruginosa* | 20(10) | 16.13(8.06) |
| *Escherichia coli* | 22(7) | 17.74(5.64) |
| *Staphylococcus. aureus* | 7(3) | 5.65(2.43) |
| *Acinetobacter baumannii* | 7(2) | 5.65(1.61) |
| *Staphylococcus epidermidis* | 6(4) | 4.84(3.23) |
| *Staphylococcus capitis* | 9(0) | 7.26 |
| *Streptococcus pyogenes* | 7(0) | 5.65 |
| *Streplococcus agalactiae* | 6(0) | 4.84 |
| *Enterococcus faecalis* | 5(2) | 4.03(1.61) |
| *Salmonella typhimurium* | 5(0) | 4.03 |
| *Klebsiella. pneumoniae* | 16(4) | 12.90(3.23) |
| *Enterobacter cloacae* | 5(0) | 4.03 |
| *Stenotrophomonas maltophilia* | 5(0) | 4.03 |
| *Proteus mirabilis* | **2**(0) | 1.61 |
| *Streptococcus pneumonia* | 2(0) | 1.61 |
| Total | 124(32) | 100.00(25.81) |
