# Supplementary material for: Multiple cross displacement amplification-a more applicable technique in detecting Pseudomonas aeruginosa of ventilator-associated pneumonia (VAP)
Source: Crit Care. 2020 Jun 8;24:306. doi: 10.1186/s13054-020-03003-4 (PMC7276953; doi:10.1186/s13054-020-03003-4)
Supplement: Supplementary file 2 — Additional file 2. Bacterial strains list of BALF by standard culture. [file 13054_2020_3003_MOESM2_ESM.docx]

### Additional file 2 –Bacterial strains list of BALF by standard culture

| **Bacteria** | **Strains (MDR)** | **Frequency (%)** |
| --- | --- | --- |
| *Pseudomonas. aeruginosa* | 26(10) | 27.66（10.63） |
| *Acinetobacter baumannii* | 16(4) | 17.02（4.26） |
| *Escherichia coli* | 10(2) | 10.64（2.13） |
| *Klebsiella. Pneumonia* | 10(2) | 10.64（2.13） |
| *Stenotrophomonas maltophilia* | 6(0) | 6.38 |
| *Enterobacter cloacae* | 5(0) | 5.32 |
| *Proteus mirabilis* | 3(0) | 3.19 |
| *Burkholderia cepacia* | 2(0) | 2.13 |
| *Staphylococcus. aureus* | 8(2) | 8.51（2.13） |
| *Enterococcus faecalis* | 5(0) | 5.32 |
| *Staphylococcus epidermidis* | 2(0) | 2.13 |
| *Streptococcus pneumonia* | 1(0) | 1.11 |
| Total | 94(20) | 100（21.28） |
